# Targeted killing of colorectal cancer cell lines by a humanised IgG1 monoclonal antibody that binds to membrane-bound carcinoembryonic antigen

**DOI:** 10.1038/sj.bjc.6604289

**Published:** 2008-03-18

**Authors:** P J Conaghan, S Q Ashraf, M G Tytherleigh, J L Wilding, E Tchilian, D Bicknell, N JMcC Mortensen, W F Bodmer

**Affiliations:** 1 Cancer Research UK, Cancer & Immunogenetics Laboratory, Weatherall Institute of Molecular Medicine, Headington, Oxford, UK; 2 Department of Colorectal Surgery, John Radcliffe Hospital, Headington, Oxford, UK

**Keywords:** PR1A3, CEA, ADCC, colorectal cancer

## Abstract

The distribution of carcinoembryonic antigen (CEA) in colorectal cancer (CRC) differs from that in normal colorectal tissue, being found on all borders of the cell membrane and hence enabling access to intravenous antibody, making CEA a good target for antibody-based therapy. The distinctive anti-CEA antibody, PR1A3, binds only membrane-bound CEA. Humanised PR1A3 (hPR1A3) was assessed both *in vitro* cytotoxicity and binding assays with colorectal cancer cell lines expressing varying levels of CEA. Human peripheral blood mononuclear cells (PBMCs) and purified natural killer (NK) cells were used as effectors. The *in vitro* assays demonstrated hPR1A3 CEA-specific binding and antibody-dependent and CEA-specific killing of human colorectal cancer cell lines by human PBMCs. The effect increased with increasing concentration of antibody and surface CEA, and was lost by using the parent murine IgG1 PR1A3. Killing was also blocked by antibody to the Fc-*γ*IIIA receptor. Purified human NK cells were effective at much lower effector:target ratios than unfractionated PBMCs, indicating that NK cells were the main mediators of hPR1A3-based CEA-specific killing. The results support the development of hPR1A3 for therapy of colorectal cancer.

There has been a recent resurgence of interest in the use of monoclonal antibodies for the treatment of cancer, mostly in the setting of metastatic disease, and already a number of antibodies have been licensed for use (for review see Reichert and Valge-Archer, 2007). Antibody therapy directed at cell surface components, in particular cell membrane receptors, has the potential to exploit the specificity and sensitivity of the immune system's ability to recognise cell membrane receptors, and so to achieve selective therapeutic effects without the toxicity of standard chemotherapy. As a result antibody therapy has become a useful adjunct to the treatment of cancer. This comes despite poor initial results with murine antibodies (Clynes, 2006). The development of chimeric and humanised antibodies has led to the development of a second generation of antibodies that hardly, if at all, stimulate the development of human anti-mouse antibodies, and which are potent activators of the immune system (Carter, 2006). Currently, about 19 antibodies are licensed for clinical use, 11 of which are for the treatment of cancer (Reichert and Valge-Archer, 2007). There are, it is estimated, at least a further 150 antibodies in development. Antibody use has had a major impact on the treatment of haematological malignancies with excellent response rates seen using rituximab (anti-CD20) in follicular and B cell non-Hodgkin's Lymphoma (Olszewski and Grossbard, 2004). Their impact in solid tumours has, however, been less dramatic. A major reason for this must be the limited penetration of antibodies, being large glycoproteins, into the tumour. Three antibodies have been licensed by the US FDA for use in advanced colorectal cancer. These include antibodies against EGFR (cetuximab) and VEGF (bevacizumab). A problem with the former is the presence of EGFR on cells of a variety of normal tissues leading to a degree of nonspecificity with respect to cancer. The EGFR antibody also appears to be effective in only a proportion of cancer patients. It is therefore important to look for other potential antigens to use as targets to broaden the number of patients for which antibody-based immunotherapy may be effective.

Carcinoembryonic antigen (CEA or CEACAM5) was first recognised as a potential human tumour-specific antigen in the 1960s (Gold and Freedman, 1965; Berinstein, 2002). CEA has characteristics that make it a useful target for antibody therapy in colorectal cancer (CRC). It is overexpressed in the vast majority of CRCs (Chan and Stanners, 2007). More importantly, however it is always aberrantly expressed in CRC. While the CEA expressed on normal colonic epithelium is inaccessible to IgG antibody, being found only on the luminal surface of the cell, this expression pattern changes in the neoplastic cell so that CEA is additionally expressed on the basal and lateral membranes (Hammarstrom, 1999) making it accessible to blood-borne antibody. Although a member of the Ig superfamily, CEA is linked to the cell membrane by a glycophosphatidylinositol (GPI) anchor (Thompson *et al*, 1991) and thus has no direct intracellular signaling motif. The functional significance, if any, of its overexpression in colorectal cancer remains unclear. The biggest disadvantage of CEA as a target is that it is readily cleaved from the cell surface and so shed into the blood stream from tumours, either directly or via the lymphatics, which is why the level of serum CEA has been used in the clinic as a marker for screening and recurrence, especially of colorectal cancer (Chau *et al*, 2004). Serum CEA binds to most of the currently used anti-CEA antibodies hindering them from reaching their target and so largely mitigating against any potential clinical effect. This is, however, not the case for the murine antibody, mPR1A3 developed in the ICRF laboratories in London by Richman and Bodmer (1987).

PR1A3 was shown to target the B3 domain and GPI anchor of the CEA molecule by Durbin *et al*, 1994 (see Figure 1), and was subsequently humanised by Stewart *et al*, 1999. Murine PR1A3 has been shown to react poorly with soluble CEA, which lacks the GPI anchor, and has been used in immunoscintigraphy for the detection of colorectal tumours with a high degree of specificity (Granowska *et al*, 1989). In contrast to EGFR and VEGF, there are so far no unconjugated, or ‘naked’ antibodies to CEA being used for the treatment of colorectal cancer. The anti-CEA antibodies that are currently used in pilot trials, are administered as radioconjugates (Wong *et al*, 2004; Liersch *et al*, 2007).

Strong evidence for the suggestion that the antitumour effects of antibodies are mediated mainly by ADCC (antibody-dependent cellular cytotoxicity) comes from mouse knockout studies which showed that antibody antitumour effects were largely absent in mice lacking the appropriate Fc receptor (Clynes *et al*, 1998). In humans, natural killer (NK) cells that express the CD16 Fc*γ*receptor are thought to be the main cell type that could mediate ADCC of tumours (Arnould *et al*, 2006). Several criteria, therefore, need to be fulfilled before an antibody can be considered for therapy (Reichert and Valge-Archer, 2007). Humanised antibodies are more potent activators of the human immune system than their murine equivalents. Internalisation of antibody-antigen complexes, leading to a loss of antibody complexes available for binding to Fc receptor-bearing cells, will diminish the effectiveness of antibody-based killing.

This study has three aims. First to analyse the ability of humanised hPR1A3 to react with a panel of colorectal cancer cell lines expressing high and low levels of CEA. Second to explore the use of hPR1A3 in an *in vitro* cytotoxicity model and to compare human PBMC with partially purified NK cells as effectors for ADCC. The third aim was to investigate whether soluble CEA inhibited the ADCC activity of hPR1A3 *in vitro*.

## MATERIALS AND METHODS

### Cell lines

The gastric carcinoma cell line MKN45 (Motoyama *et al*, 1979) was obtained from Cell Services LIF, Cancer Research UK. All other cell lines were colorectal cell lines:

HCT-116 (Brattain *et al*, 1981) and SKCO-1 (Fogh, 1975) were originally obtained from ATCC; GP5d (Solic *et al*, 1995) and HT55(Watkins and Sanger, 1977) were obtained from ECACC; LS174T (Tom *et al*, 1976) was obtained from BH Tom, NW University Med Centre, Chicago, Ill, USA, PC/JW (Paraskeva *et al*, 1984) was obtained from C. Paraskeva, Directors Lab, CRUK, London and C70 (Browning *et al*, 1993) was established in the Cancer and Immunogenetics Laboratory.

All cell lines were maintained in culture in Dulbecco's modified Eagle's (E4) medium with 1% L-glutamine, 10% fetal calf serum (FCS) and 1% penicillin/streptomycin. They were incubated at 37°C in a humidified 10% CO_2_ environment. For the chromium-release and EuTDA assays the cells were suspended in 2% RPMI-1640 medium with 1% glutamine and 10% FCS.

### Peripheral blood mononuclear cells

Peripheral blood mononuclear cells (PBMCs) were isolated either from fresh whole blood from healthy laboratory volunteers, having taken informed consent, or from leucodepletion filters obtained from single donors following blood donation (Courtesy of Cristina Navarrete, Colindale National Blood Service, London, UK). White cells were eluted from the filter using 5 mM ethylenediaminetetraacetic acid. Fresh whole blood was mixed with an equal volume of an RPMI-1640/citrate solution (40 ml 3.3% sodium citrate, 2 ml 5 mM 2-mercaptoethanol, 200 ml RPMI-1640+hepes) as an anticoagulant.

Both sources of PBMC were then processed in an identical fashion. Following Ficoll/hypaque density centrifugation, the PBMC layer was withdrawn from the interface and washed once with RPMI-1640 to remove excess Ficoll, spinning at 800 **g** (Boyum, 1968) and then a second time, spinning at 200 **g** for 10 min to remove platelets. The resulting PBMCs were resuspended in RPMI-1640/10% FCS/1% glutamine (complete RPMI-1640), kept at room temperature and used within 12 h of preparation.

### Antibodies

#### PR1A3

The original is a murine IgG1*κ* monoclonal antibody to CEA (Richman and Bodmer, 1987) that was later humanised (Stewart *et al*, 1999). Both murine (mPR1A3) and humanised (hPR1A3) antibodies were obtained from the Biotherapeutics Development Unit, Clare Hall, Cancer Research UK, London, UK.

#### Anti-CD16

Two different clones of this antibody were used: MEM154 (Biovendor Laboratory Medicine Inc.) and 3G8 (BD Biosciences, Pharmingen, USA). Both are murine monoclonal IgG1*κ* antibodies against the human Fc*γ*IIIA receptor (CD16A; FCGR3A). F(ab’)_2_ derived from the 3G8 clone was obtained from Ancell Corp, Bayport, MN, USA.

#### Anti-prostate specific membrane antigen

Murine monoclonal antibody 107-1A4 to PSMA was kindly provided by Robert Vessella (Univ Washington, USA).

#### Polyclonal rabbit anti-mouse antibody

This was obtained from DAKO A/S, Denmark, and was diluted to 1 : 100 in RPMI-1640/1% FCS.

#### Anti-*β*-galactosidase antibody

clone 4C7 (Durbin and Bodmer, 1987), was obtained from the Monoclonal Antibody Service, Clare Hall, Cancer Research UK, London, UK, and used at a final concentration of 0.4 *μ*g ml^−1^ in RPMI-1640/1% FCS.

#### AUA-1

This was obtained from the Monoclonal Antibody Service, Clare Hall, Cancer Research UK, London, UK (Epenetos *et al*, 1982). It has been shown to be an anti-EpCam antibody (Spurr *et al*, 1986).

#### FITC-conjugated murine anti-human IgG and FITC-conjugated rabbit anti-mouse IgG

These were obtained from Sigma-Aldrich, Poole, UK.

#### CD3-FITC, CD16-PE, CD56-APC, IgG1-FITC (isotype control), IgG1-PE (isotype control) and IgG1-APC (isotype control)

These were obtained from BD Biosciences Pharmingen, Oxford, UK.

### Chromium-release cytotoxicity assay

Peripheral blood mononuclear cells were prepared as above and suspended at a concentration of 1 × 10^7^ ml^−1^ in RPMI-1640 supplemented with 10% FCS and 1% glutamine (complete medium). 2 × 10^6^ target cells were centrifuged at 400 **g** for 5 min, decanted and labelled by resuspending the pellet in 0.1 ml of 7.4MBq of Na_2_^51^CrO_4_. This cell suspension was incubated at 37°C for 60–100 min depending on the optimal labelling time for the particular cell line. Optimal labelling time had previously been determined by choosing the labelling time with the highest maximal lysis:background radioactivity ratio. The cells were then washed twice with RPMI-1640 and suspended in complete RPMI-1640 at a concentration of 1 × 10^5^ cells per ml. Target cells (100 *μ*l (1 × 10^4^)) and PBMCs (100 *μ*l (1 × 10^6^)) were added to microcentrifuge tubes to give an effector: target ratio of 100 : 1. Various antibody concentrations (20 *μ*l) were then added to the relevant tubes. Triton (120 *μ*l of 5%) was added to 100 *μ*l of target cells to obtain maximum release values. All tubes were made up to the same volume using complete RPMI-1640. The tubes were spun at 200 **g** for 2 min and the pellet of combined target and effector cells was incubated at 37°C for 4 h in the presence of antibody. The tubes were then spun at 200 **g** for 5 min and 35 *μ*l of the supernatant added to 100 ul Optiphase Supermix (Perkin Elmer, Boston, MA, USA) in a 96-well plate. The ^51^Cr concentration in each well was then determined using a Microbeta plate reader.

### Fluorescence-based EuTDA cytotoxicity assay

Five microlitres of BATDA (Blomberg *et al*, 1996) (Perkin Elmer, Boston, MA, USA) were added to 2 × 10^6^ target cells suspended in complete RPMI-1640 and incubated for 10–25 min at 37°C depending on the optimal labeling time for the particular cell line, determined as described for the ^51^Cr-release assays. The relative concentrations of target cells and antibody in the microcentrifuge tubes were similar to those used for the ^51^Cr release assay. However, varying effector:target cell ratios were used for the fluorescence-based assay. After spinning at 200 **g** for 2 min the tubes were incubated at 37°C for 2 h and then spun again at 200 **g** for 5 min. A total of 20 *μ*l of the resulting supernatant was added to 200 *μ*l of Europium in black 96-well plates and the resulting fluorescence was then read in a time-resolved fluorometer using an excitation wavelength of 340 nm and an emission wavelength of 615 nm.

### CEA ELISA assay

CEA levels were determined using a *β*-galactosidase/anti-*β*-galactosidase ELISA (Durbin and Bodmer, 1987). Cells were plated onto a poly-L-lysine coated 96-well Nunc-Immuno PolySorp plate at a concentration of 2.5 × 10^4^ cells per well. Murine PR1A3 was used as the CEA-detecting antibody with rabbit anti-mouse antiserum as the secondary antibody. The GAG complex of *β*-galactosidase with anti-*β*-galactosidase antibody was made by dissolving *β*-galactosidase (*E.coli β*-galactosidase lyophilised powder; Sigma-Aldrich, Poole, Dorset, UK) at a concentration of 500 U ml^−1^ in 100 mM TRIS/100 mM MgCl_2_/100 mM 2-mercaptoethanol with 300 *μ*g ml^−1^ of 4C7 and incubating this complex at 4°C overnight. The complex so formed was then added at a dilution of 1 : 750 in RPMI-1640/1% FCS. The GAG complex binds to free antigen-binding sites on the rabbit anti-mouse IgG antibody, which is already bound to the PR1A3 attached to the CEA on the cells. The substrate 4-methylumbelliferyl-B-D-galactoside (Sigma-Aldrich, Poole, Dorset, UK) was first prepared at a concentration of approximately 0.3 mg ml^−1^ in a buffer of 1 mM MgCl_2_/100 mM 2-mercaptoethanol . After stirring for 30 min, the solution was filtered to remove excess substrate. This substrate solution was added to each well to start the reaction. After incubation for 40 min in the dark at room temperature, fluorescence was measured using an excitation wavelength of 365 nm and an emission wavelength of 445 nm.

### Flow cytometric analysis of CEA internalisation

The concentration of cells to be analysed was adjusted to 1 × 10^6^ ml^−1^ and the cells washed once with phosphate-buffered saline (PBSA) containing 2% FCS and then centrifuged at 400 **g** for five minutes. The resulting pellet was resuspended in cold PBSA containing 2% FCS and incubated on ice with either 100 *μ*l of hPR1A3 at 20 *μ*g ml^−1^, 100 *μ*l of AUA-1 at 15 *μ*g ml^−1^ or a medium control for 30 min. The cells were next washed again, with PBSA containing 2% FCS and incubated in a water bath at 37°C for 0, 1, 2 or 3 h. The cells were then incubated with a 1 : 50 dilution of FITC-conjugated anti-human-IgG for hPR1A3 and a 1 : 100 dilution of FITC-conjugated anti-murine IgG for AUA-1 on ice in the dark for 30 min before being washed again and resuspended in PBSA containing 2% FCS. The cells were then passed through a fluorescence-activated cell sorting (FACS) Calibur flow cytometer and the results analysed using CellQuest software.

### NK cell enrichment

Fresh PBMC or eluted PBMC from leucodepletion filters were enriched for NK cells using a Human NK Cell isolation kit (Miltenyi Biotec) following the manufacturer's instructions. This involved adding the NK cell Biotin-Antibody cocktail (10 *μ*l per 10^7^ cells) which contained antibodies against T cells, B cells, stem cells, dendritic cells, monocytes, granulocytes and erythroid cells. Following incubation for 10 min at 4°C, the NK Cell magnetic Microbead Cocktail was added. This was left for 15 min at 4°C and the cells then washed with MACS buffer (PBSA/0.5% FCS/2 mM EDTA) and centrifuged. The cell pellet was resupended in 0.5 mls MACS buffer and then passed through an LS magnetic column to remove cells that bound the antibodies in the cocktail. The cells that passed through were then collected and suspended in RPMI-1640 with and without IL-2 (Peprotech) at 10 ng ml^−1^ and incubated overnight at 37°C. Aliquots pre and post sorting were taken for FACS analysis with the antibodies CD3, CD56 and CD16 and the appropriate isotype controls. Thus, 1 × 10^6^ cells were suspended in 2 ml of FACS buffer (PBSA/1%FCS/1% sodium azide) and centrifuged. The supernatant was removed and the cells resuspended in the residual buffer. The antibodies were then added and left for 20 min at 4°C before washing and then fixing with 300 *μ*l of 2% paraformaldehyde. The fixed cells were analysed on a FACS Calibur flow cytometer. Gating of the lymphocyte population in the forward versus side scatter plot revealed a purity of at least 85% with respect to CD16 and CD56 binding. The resulting NK cells were used in cytotoxicity assays with hPR1A3 against SKCO-1, which is known to express CEA.

### Effect of soluble CEA on PR1A3-induced ADCC

Purified CEA, obtained from human liver colorectal metastasis, was purchased from Chemicon and diluted in RPMI-1640 for use in ADCC assays with NK cells (CD3−/CD56+/CD16+) that were isolated from PBMC from a healthy volunteer. CEA was added to achieve final concentrations of 2 and10 *μ*g ml^−1^. This concentration far exceeds any concentration that would be found in the serum of a colorectal cancer patient. A level above 5 ng ml^−1^ is generally accepted as being raised. Patients with CEA levels above 15 ng ml^−1^ have been found to have a worse prognosis (Wiratkapun *et al*, 2001). The cytotoxicity assay was then carried out as described above.

### FACS analysis for competitive inhibition of hPR1A3 binding in the presence of soluble CEA

Soluble CEA at a final concentration of 10 *μ*g ml^−1^ was added to hPR1A3 (final concentration 20 *μ*g ml^−1^). The mixture was incubated in a 1.7 ml eppendorf microtube for 45 min at room temperature. Humanised PR1A3 alone, at a concentration of 20 *μ*g ml^−1^, and medium alone were similarly incubated in a microtube. Cells to be assayed with these various mixtures were adjusted to a concentration of 1 × 10^6^ ml^−1^ in RPMI-1640 complete medium and washed with FACS buffer (PBSA/1%FCS/1% sodium azide). The resulting pellet was resuspended in 10 *μ*l FACS buffer and then incubated on ice with 100 *μ*l 20 *μ*g ml^−1^ mPR1A3, hPR1A3/CEA (as prepared above) or medium control for 30 min. The cells were then washed twice with FACS buffer and incubated on ice in the dark for 30 min with a 1 : 50 dilution of FITC-conjugated anti-human-IgG (for hPR1A3). The resulting labelled cells were washed again with 2 ml of FACS buffer and centrifuged at 400 **g**. The supernatant was removed completely and the cells resuspended in 300 *μ*l of 2% paraformadehyde in PBSA. The labelled cells were analysed using a FACS Calibur flow cytometer as described before.

### Data analysis

Percentage lysis of the cell lines in the cytotoxicity assays was calculated as (experimental release−background release)/(maximum release−background release) × 100. Percentage specific lysis was calculated as (experimental release−antibody independent release)/(maximum release−antibody independent release) × 100. The s.e.m. of multiple experiments was calculated using Graphpad Prism software, San Diego, CA, USA. Standard normal distribution tests were used to assess the significance of the differences found.

## RESULTS

### hPR1A3 binds to membrane-bound CEA and the antibody–antigen complex is not internalised after 3 h

**Figure 2:**
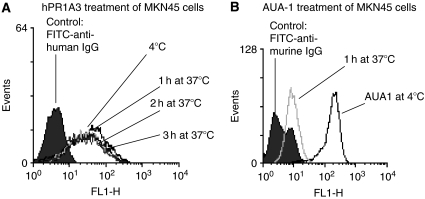
Time-course FACS analysis of hPR1A3 binding to CEA-expressing MKN45 cells. The cell line was labeled with primary antibody (PR1A3 or AUA-1) at 4°C and then incubated for varying times at 37°C before addition of secondary antibody (FITC-conjugated anti-human IgG or anti-mouse IgG respectively). (**A**) Analysis over time suggests that the CEA-PR1A3 complex is not internalised within 3 h of binding to CEA. (**B**) Binding of the monoclonal antibody AUA-1 to EpCAM on the surface of MKN45 cells leads to internalisation within one hour.

Humanised PR1A3 showed specific binding to MKN45, a high CEA-expressor. After three hours incubation of MKN45 at 37°C with hPR1A3, no change was observed in the amount of antibody detected on the cell surface (Figure 2A). This is in contrast to the results obtained in similar experiments using the anti-EpCAM monoclonal antibody, AUA-1 (Figure 2B). In that case there is already a significant reduction in the cell surface expression of EpCAM after incubation for 1 h at 37°C indicating a fairly rapid internalisation of the surface EpCAM/anti-EpCAM complex. These data show that the CEA/anti-CEA(PR1A3) complex is not significantly internalised even after 3 h of incubation at 37°C (Figure 2).

### hPR1A3 causes dose-dependent lysis of the high CEA-expressing cell line, MKN45

**Figure 3:**
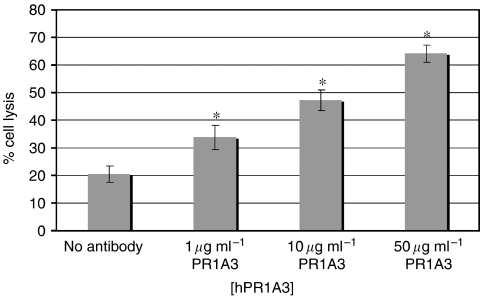
Humanised PR1A3-induced ADCC-mediated lysis is dependent on the concentration of antibody. Cr-release ADCC assays were done using MKN45 as CEA-expressing target cells and human PBMC as effectors at a ratio of 100 : 1 effectors to targets. Columns represent mean % lysis without antibody and with varying concentrations of hPR1A3. These data shown are from the analysis of 14 separate experiments using MKN45 as the target cell and with triplicate wells for each condition in each experiment. Error bars indicate standard error of the mean (s.e.m.). ^*^Indicates that significance of values is *P*<0.05 compared with samples given no antibody.

MKN45 was used as a high CEA expressing cell line in cellular cytotoxicity assays with ^51^Cr using different antibody concentrations. In the absence of antibody, PBMCs effected a low but significant level of spontaneous killing. The level of killing increased with increasing concentrations of antibody, as would be expected for ADCC (Figure 3).

### Colorectal cancer cell lines express varying levels of CEA

The level of CEA expression on a subset of cell lines was assessed using an ELISA assay with mPR1A3, and is given in Table 1 as mean arbitrary fluorescence units (±s.d.).

These data correlate well with those obtained from FACS analysis, RT–PCR and micro-array expression data (see Supplementary Figure 1).

### The level of hPR1A3-mediated ADCC depends on the level of CEA expression

**Figure 4:**
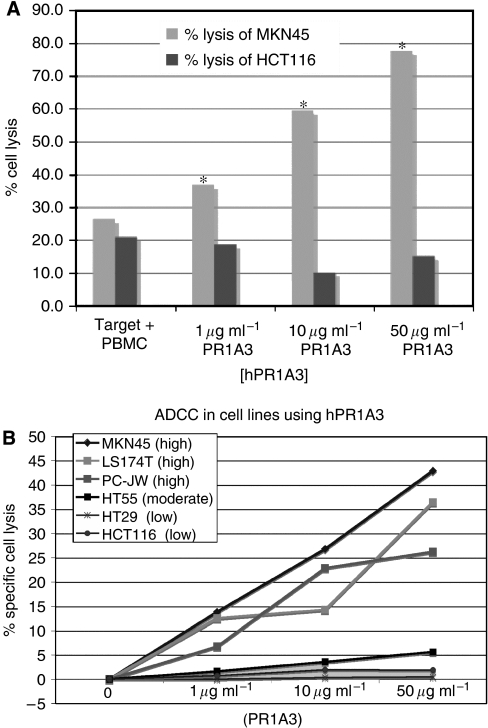
hPR1A3-mediated ADCC lysis of colorectal cancer cell lines depends on their level of CEA expression. (**A**) Effect of increasing concentrations of hPR1A3 on lysis of CEA-positive (MKN45) and -negative (HCT-116) cell lines. Fluorescence-based ADCC assays were done using human PBMCs as effector cells in a ratio of 100 : 1 with target cells. Columns represent % lysis in the presence of both target and effector cells with no, or with increasing concentrations of hPR1A3. ^*^P<0.05 comparing the cell lysis between MKN45 and HCT116. (**B**) Comparison of hPR1A3-mediated ADCC based lysis in cell lines with different levels of CEA expression (shown in parentheses) based on results in Table 1. The EuTDA-based ADCC assay was done using human PBMCs as effector cells at ratios of 100 : 1 with the various target cell lines. Nonspecific spontaneous killing levels have been subtracted to reflect antibody-specific lysis only.

HCT-116 was identified by FACS, ELISA and RT–PCR assays to have no or at most a very low level of CEA expression. The killing of the high expressing cell line MKN45 was therefore compared with that of HCT-116 by hPR1A3 in the presence of human PBMC using a EuTDA assay. While MKN45 was killed as expected, there was no increase in lysis of HCT-116 above spontaneous killing even with the highest concentration of antibody (Figure 4A). The variation in hPR1A3-based ADCC lysis between cell lines expressing different levels of CEA was assessed using PBMC and a EuTDA assay. The results shown in Figure 4B indicate a good correlation between CEA levels and the degree of hPR1A3-mediated killing (Figure 4).

### hPR1A3-mediated ADCC-based killings depends on the Fc portion of the antibody

**Figure 5:**
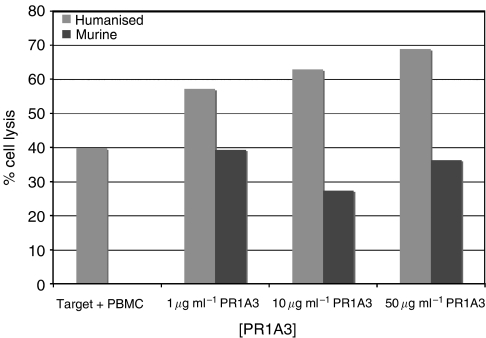
Comparison of humanised IgG1 and murine IgG1 isotypes of PR1A3 in fluorescence-based ADCC assays using human PBMCs as effectors and the MKN45 cell line. Effector:target ratios of 100 : 1 were used in all assays. Columns represent mean % lysis from triplicate wells containing both target and effector cells with no, or with increasing concentrations of hPR1A3.

Since the murine IgG1 isoform does not associate strongly with the human Fc*γ*IIIA receptor (Lubeck *et al*, 1985), mPR1A3 was compared with hPR1A3 in killing assays on MKN45. The results (Figure 5) show that murine PR1A3 did not kill above background, in contrast to hPR1A3. This adds to the support for the specificity of hPR1A3-based killing and suggests its dependence on appropriate interaction with an Fc*γ* receptor (Figure 5).

### hPR1A3-dependent and spontaneous killing are both inhibited by an anti-CD16 antibody, but only antibody-dependent killing is inhibited by an F(ab’)_2_ of anti-CD16

**Figure 6:**
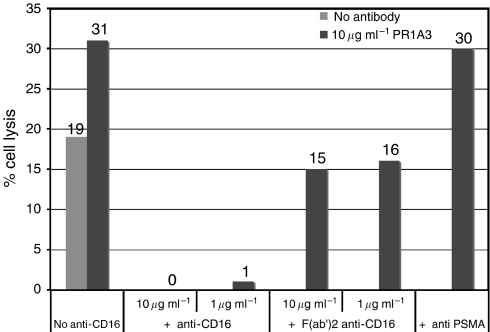
Effect of whole or F(ab’)_2_ anti-CD16 on hPR1A3-mediated lysis. Fluorescence-based ADCC assays were done using human PBMCs as effector cells in a ratio of 100 : 1 with MKN45 target cells. Columns represent mean % lysis from triplicate wells containing both target and effector cells ±10 *μ*g ml^−1^ hPR1A3 ±second antibody as indicated. The anti-CD16 constructs and anti-PSMA control were added at the same time as the effector cells without prior incubation.

Since the NK effector cells in PBMC, which are presumed to mediate the majority of antibody-dependent killing, do so via the CD16 (Fc*γ*IIIA) receptor (Titus *et al*, 1987; Lanier *et al*, 1988; Moretta *et al*, 1989), the blocking effects on hPR1A3 killing of MKN45 by anti-CD 16 and a F(ab’)_2_ of the same antibody were investigated (Figure 6).

The results of blocking experiments with two different concentrations of the reagents are shown in Figure 6. These data show that anti CD16 completely blocked both spontaneous and hPR1A3-induced killing. To ensure that this was a direct effect of blocking the CD16 antibody receptor and not simply a consequence of adding a second antibody which might compete for Fc-receptor sites, we added a non-specific antibody against prostate membrane-specific antigen instead of anti-CD16 and this did not block hPR1A3-mediated killing of MKN45.

In marked contrast to the blocking effects of whole CD16 antibody, the F(ab’)_2_ of the anti-CD16, which lacks the Fc-portion of the antibody required for binding to the CD16 receptor on effector cells, abolished only antibody-dependent killing but did not affect the spontaneous lysis.

### Purified NK cells are able to elicit ADCC with hPR1A3 at much lower effector:target ratios than are unfractionated PBMC

To establish that the major killing effect seen with hPR1A3 was actually due to NK cells as conventionally defined, purified NK cells were used in assays of hPR1A3 killing of the high CEA expressing colorectal cell line, SKCO-1. NK cells were enriched from PBMCs obtained from buffy coat (NBS) using the Human NK Isolation kit, as described in Materials and Methods.

**Figure 7:**
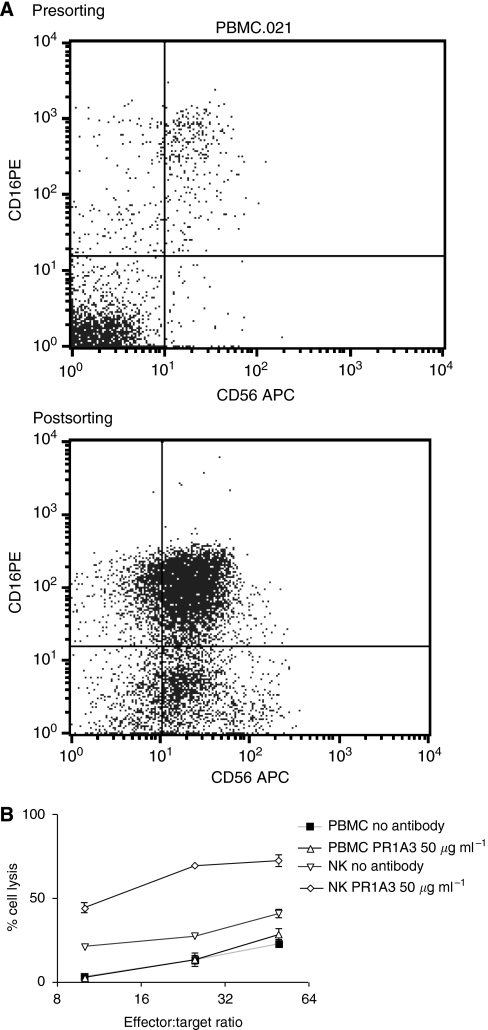
(**A**) Demonstration of NK cell purification. NK cells were purified from a single donor buffy coat sample (NBS) using the Human NK isolation kit. FACS analysis was performed using CD56 and CD16 antibodies pre- and post-sorting to show the enrichment of NK cells, which are both CD16- and CD56-positive. (**B**) Purified NK cells are much more effective killers than unfractionated PBMC. PBMC and NK cells were isolated from a single donor buffy coat (NBS). SKCO-1 was used as the target in the presence of PBMC (△) or NK cells (◊) with PR1A3 (50 *μ*g ml^−1^), or with no antibody (PBMC, ▪; NK, ▽). The flouresence-based EuTDA assay was used with effector:target ratios ranging from 10 : 1 to 50 : 1.

The extent of enrichment for NK cells is illustrated by the FACS analysis shown in Figure 7A, using antibodies to CD16 and CD56. The second panel clearly documents the extensive purification of the CD16 and CD56 positive NK cells. The results of ADCC assays with hPR1A3 using either these purified NK cells or unfractionated PBMCs as effectors, and SKCO-1 as target cells, are shown in Figure 7B. The difference in the effectiveness of killing by NK cells as compared to unfractionated PBMCs is striking. Whereas in both cases there is some spontaneous killing in the absence of antibody, the NK cell antibody-specific killing is already very clear at the lowest effector:target ratio of 10. There is, on the other hand, barely detectable specific killing with the PMBCs even at a 50 : 1 effector: target ratio. Further data (see Supplementary Figure 2) demonstrate that ADCC can occur in the presence of purified NK cells even at effector: target ratios as low as 1 : 1. These results provide strong support for the assumption that the hPR1A3 antibody-specific killing of CEA expressing human cell lines is almost entirely due to the action of the human NK cells as effectors.

### Neither hPR1A3 binding to, nor hPR1A3-mediated killing of MKN45 are blocked by soluble CEA

**Figure 8:**
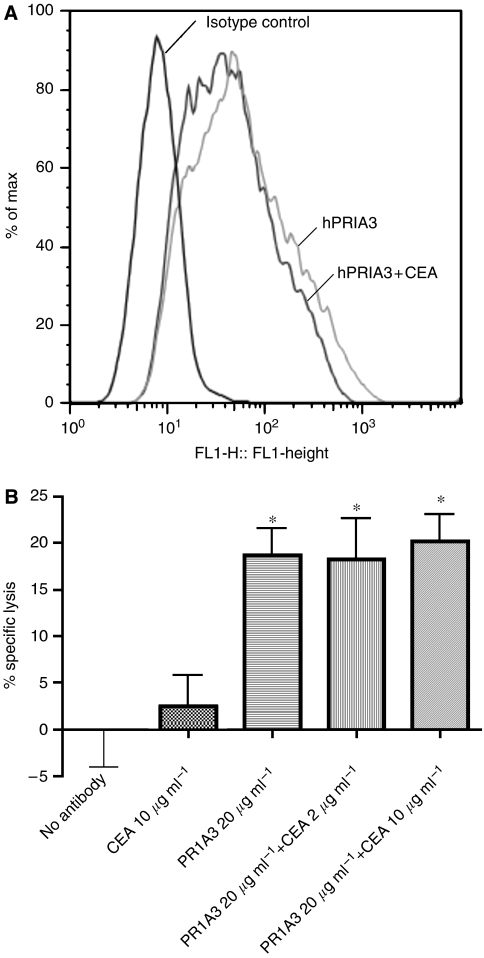
(**A**) Indirect staining and FACS analysis to study the effect of addition of soluble CEA on humanised PR1A3 binding to SKCO-1. The left histogram plot is the isotype control. The light grey curve represents the histogram curve for hPR1A3 only (20 *μ*g ml^−1^) and the dark grey curve represents the curve for hPR1A3 (20 *μ*g ml^−1^) that has been preincubated with 10 *μ*g ml^−1^ of soluble CEA. (**B**) ADCC assay with hPR1A3 on its own, or after preincubation with soluble CEA at final concentrations of 2 and 10 *μ*g ml^−1^, using purified NK cells (CD56+/CD16+/CD3−) as effectors and fluorescently labelled SKCO-1 cells as targets (effector:target ratio used was 10 : 1). The controls used were no antibody and purified CEA with target and effector cells only. ^*^Indicates *P*<0.05 compared with the specific lysis of target and effector cells alone.

The binding to the high expressing cell line SKCO-1 of hPR1A3 (20 *μ*g ml^−1^) on its own, or after preincubation with soluble CEA (10 *μ*g ml^−1^) was investigated using a FACS analysis. The results, illustrated in Figure 8A, show that there is no reduction in reactivity of hPR1A3 with MKN45 after preincubation with CEA, as compared to hPR1A3 alone. This corroborates for hPR1A3 the earlier results obtained by Durbin *et al*, 1994 with mPR1A3, which showed that soluble CEA does not block this antibody's binding to membrane bound CEA (Figure 8).

To show the same lack of effect of soluble CEA on hPR1A3-specific killing, ADCC assays were carried out using fresh blood-derived NK cells and SKCO-1 as target cells with hPR1A3 on its own, or after preincubation with soluble CEA. The results, illustrated in Figure 8B, clearly show that even preincubation with 10 *μ*g ml^−1^ of soluble CEA does not reduce the specific killing effect of hPR1A3.

## DISCUSSION

Our study has clearly shown that the humanised PR1A3 antibody can be used for targeted killing of colorectal cancer lines that express cell surface CEA. Both the direct binding of the antibody to cells, and the extent of its ADCC activity against cells are dependant on the level of surface expression of CEA. The fact, as we have also shown, that CEA is not significantly internalised adds another advantage to CEA as a target for naked antibody therapy. This effectively increases the exposure times to Fc*γ* receptor-bearing cells by promoting attachment to antibody-coated target cells. We have confirmed, as was shown previously for the murine version of PR1A3, that the binding of hPR1A3 to surface bound CEA is not inhibited by soluble CEA, and in addition have shown that the same is true for its ADCC activity. This property of PR1A3 accounts for the low false-positive rate of lymph node detection in immunoscintigraphy of colorectal cancers with PR1A3 in patients (Granowska *et al*, 1989), given that it has been shown that soluble CEA drains into lymphatics and so can become sequestrated into regional lymph nodes in the absence of cancer cells (Kubo *et al*, 1992).

Human NK cells are known to express CD16 (homologous to the Fc*γ* IV receptor in mice (Nimmerjahn and Ravetch, 2005)) and are thought to play an important role in responses to antibody therapy (Liljefors *et al*, 2003). We have shown that the ADCC activity of hPR1A3 is dependent on its Fc domain. The evidence for this comes both from the fact that mPR1A3, though it binds to surface CEA on human cells, has no ADCC activity using human PBMC as effectors, and that the ADCC activity of hPR1A3 is blocked both by whole antibody to CD16 and by a CD16 F(ab’)_2_ fragment. The fact that enrichment of human NK cells can elicit hPR1A3-dependent ADCC at very low effector:target ratios when compared with unfractionated PBMC, provides strong evidence that the ADCC activity of hPR1A3 is actually mediated by human NK cells. That the addition of the complete CD16 IgG, (but not the F(ab’)_2_ fragment) also abolishes antibody independent killing may be explained either by antibody binding to CD16-bearing cells reducing their mobility in the wells due to formation of clusters of CD16+ cells or that killer cells may destroy CD16+ cells which have bound the antibody.

The affinity of hPR1A3 for CEA appears to be relatively low as reflected by the higher concentrations of PR1A3 needed to mediate ADCC as compared to cetuximab (see Supplementary Figure 2). However, this may be a potential advantage in the treatment of solid tumours, since higher affinity antibodies may penetrate less into tumours due to the majority of binding taking place at the outer most part of a tumour (Adams *et al*, 2001). Intermediate affinity antibodies may, thus, be predicted to have greater penetration into solid tumours.

Much research is now directed at conjugating antibodies with radiolabels or toxins. Although this has had some success in experimental animal models, there remain significant problems in the true *in vivo* situation, including especially the development of an immune response against the toxins or enzymes linked to a therapeutic antibody.

We suggest that the appropriateness of CEA as a therapeutic target, together with our evaluation of antibody hPR1A3's mediated ADCC activity makes this antibody a very attractive target for clinical development as a naked antibody. The main challenge may be to enhance PR1A3's ADCC activity, and this may be achieved by glycoengineering its Fc hinge region (Umana *et al*, 1999), which has been shown to be a very effective method for enhancing the effectiveness of antibody-mediated ADCC *in vitro*.

As previously discussed, only a small percentage of antibody administered intravenously actually reaches the cells of a solid tumour ((Allum *et al*, 1986; Delaloye *et al*, 1986; Epenetos *et al*, 1986; Colcher *et al*, 1987; Welt *et al*, 1990). While a small number of antibody molecules reaching their tumour target may be sufficient to elicit immune-based killing by ADCC, it seems unlikely that such small amounts of antibody reaching a tumour could have much effect in blocking function, since this would require at least the majority of the antibody's targets to be covered. This emphasises the potential importance of immune mechanisms, even for therapy with antibodies against targets such as EGFR and ErbB with known functions, and so the importance of enhancing ADCC for effective treatment, rather than improving the blocking of function. The fact that CEA has no obvious function that might be blocked by antibody does not mitigate against its use for naked antibody-based therapy on the assumption that the primary mechanism is immune and through ADCC. We believe that the results we have presented here suggest that the naked anti-CEA humanised antibody PR1A3, glycoengineered to increase its efficacy in ADCC, may be an excellent candidate for therapy of colorectal and other solid tumours that express significant levels of CEA.

## Figures and Tables

**Figure 1:**
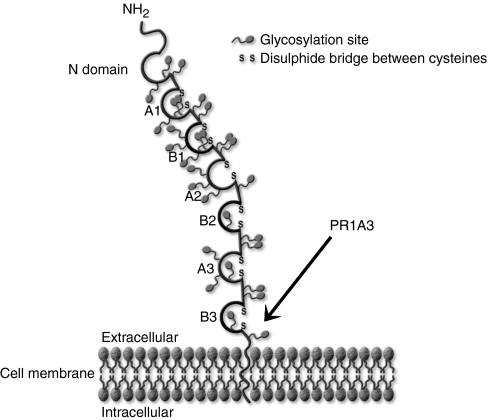
Schematic diagram showing the binding site of PR1A3 on CEA. The antibody binds at a site involving parts of the GPI anchor and the B3 domain of CEA. Access to the epitope appears to be blocked when CEA is released from the cell.

**Table 1:** CEA expression determined by GAG-ELISA and given as mean arbitrary fluorescence units ±s.d.

**Cell line**	**CEA expression (mean±s.d.)**
SKCO-1	26804 (±1322)
PC-JW	20160 (±2124)
LS174T	19833 (±2618)
MKN45	19741 (±1769)
HT55	11954 (±1667)
HT29	4688 (±1185)
HCT-116	1995 (±1035)
