# Supplementary material for: Targeted killing of colorectal cancer cell lines by a humanised IgG1 monoclonal antibody that binds to membrane-bound carcinoembryonic antigen
Source: Br J Cancer. 2008 Mar 18;98(7):1217–25. doi: 10.1038/sj.bjc.6604289 (PMC2359646; doi:10.1038/sj.bjc.6604289)

**Supplementary Information**

**Figure 1: CEACAM5 expression**

Figure1a: The graph shows the expression profile of CEACAM5 in a panel of colorectal cancer cell lines. The data were obtained from an affymetrix expression array analysis and are displayed in rank order.

Figure 1b: FACS analysis histogram profile of MKN45 and several colorectal cell lines. Control (orange), HCT116 (pale blue), HT55 (purple), HT29 (blue), C70 (green), LOVO (black) and MKN45 (red).


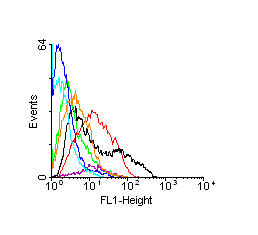


**Table 1:** RT-PCR results for CEA measured on a panel of colorectal cell lines.

| **Cell line** | **RT-PCR** |
| --- | --- |
| SKCO-1 | positive |
| LoVo | positive |
| LS411 | positive |
| HT55 | positive |
| SW403 | positive |
| SNU C2B | positive |
| GP5d | positive |
| C70 | positive |
| Car-1 | negative |
| NCI-H7176 | negative |
| HCT116 | negative |
| RKO | negative |

**Figure 2:** NK cells are potent killers of antibody-coated targets.

Using purified NK cells as effectors this graph demonstrates that ADCC occurs at effector:target ratios as low as 1:1. The NK cells were obtained from a leucodepletion filter (Colindale NBS) derived buffy coat and stimulated overnight with 10ng/ml of IL-2 prior to use in the assay. SKCO-1, a high EGF(Epidermal growth factor) receptor expressing cell line ,was used as the target in the fluorescence based EuTDA assay. Cetuximab is a widely used antibody to EGFR.


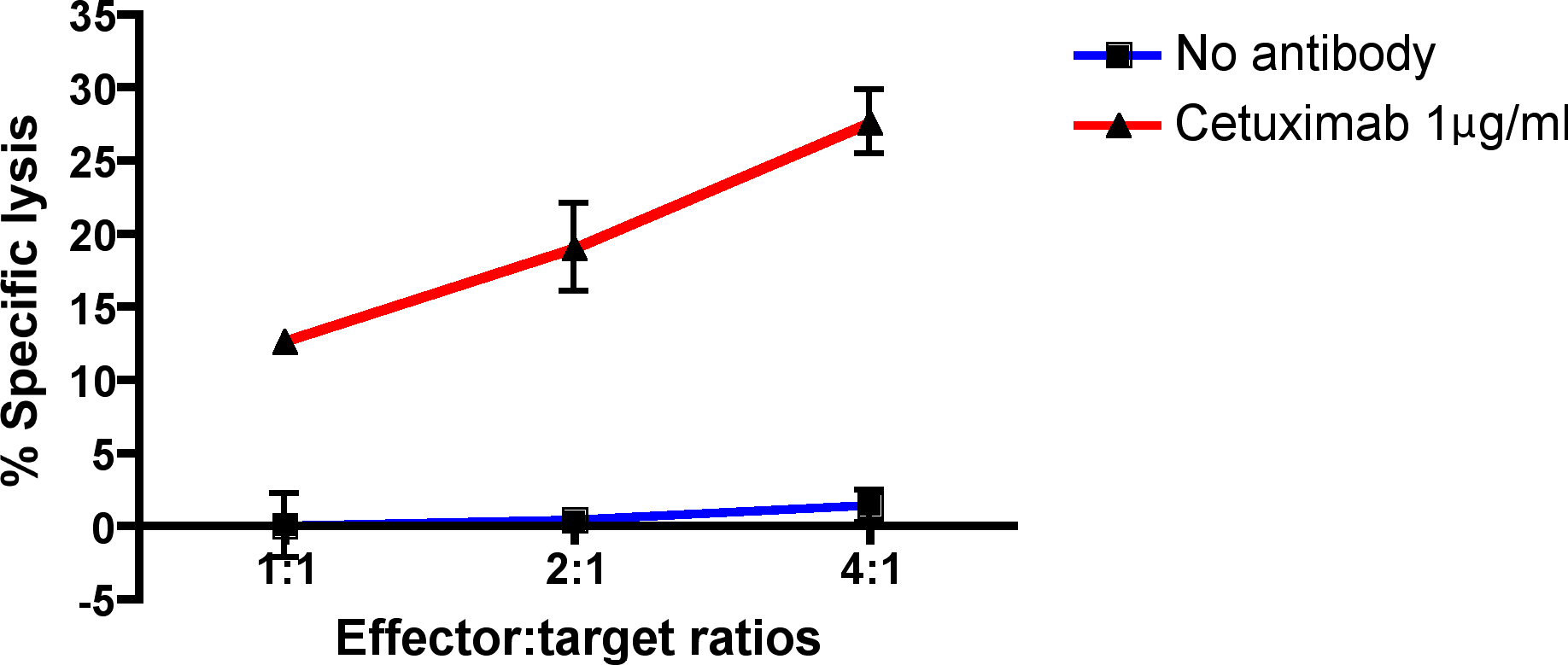

Supplement: Supplementary Information [file 6604289x1.doc]
